# A New Approach of Modified Submerged Patch Clamp Recording Reveals Interneuronal Dynamics during Epileptiform Oscillations

**DOI:** 10.3389/fnins.2016.00519

**Published:** 2016-11-09

**Authors:** Gareth Morris, Premysl Jiruska, John G. R. Jefferys, Andrew D. Powell

**Affiliations:** ^1^Neuronal Networks Group, School of Clinical and Experimental Medicine, College of Medical and Dental Sciences, University of BirminghamBirmingham, UK; ^2^Department of Developmental Epileptology, Institute of Physiology, Czech Academy of SciencesPrague, Czechia

**Keywords:** *in vitro*, membrane chamber, LFP, patch clamp, epilepsy, high frequency activity

## Abstract

**Highlights**

- Simultaneous epileptiform LFPs and single-cell activity can be recorded in the membrane chamber.
- Interneuron firing can be linked to epileptiform high frequency activity.
- Fast ripples, unique to chronic epilepsy, can be modeled in *ex vivo* tissue from TeNT-treated rats.

Traditionally, visually-guided patch clamp in brain slices using submerged recording conditions has been required to characterize the activity of individual neurons. However, due to limited oxygen availability, submerged conditions truncate fast network oscillations including epileptiform activity. Thus, it is technically challenging to study the contribution of individual identified neurons to fast network activity. The membrane chamber is a submerged-style recording chamber, modified to enhance oxygen supply to the slice, which we use to demonstrate the ability to record single-cell activity during *in vitro* epilepsy. We elicited epileptiform activity using 9 mM potassium and simultaneously recorded from fluorescently labeled interneurons. Epileptiform discharges were more reliable than in standard submerged conditions. During these synchronous discharges interneuron firing frequency increased and action potential amplitude progressively decreased. The firing of 15 interneurons was significantly correlated with epileptiform high frequency activity (HFA; ~100–500 Hz) cycles. We also recorded epileptiform activity in tissue prepared from chronically epileptic rats, treated with intrahippocampal tetanus neurotoxin. Four of these slices generated fast ripple activity, unique to chronic epilepsy. We showed the membrane chamber is a promising new *in vitro* environment facilitating patch clamp recordings in acute epilepsy models. Further, we showed that chronic epilepsy can be better modeled using *ex vivo* brain slices. These findings demonstrate that the membrane chamber facilitates previously challenging investigations into the neuronal correlates of epileptiform activity *in vitro*.

## Introduction

A major issue in studies of epilepsy is how much the activity of different types of neurons is altered during epileptiform bursts. The challenge is that recording configurations which support induction of robust epileptiform bursts (typically interface chambers) are not conducive to the visually guided patch clamp approach needed to identify and record from individually identified neurons during these bursts. In contrast, submerged slices allow visually guided patching, but generally do not support robust epileptiform activity. Thus, an approach which combines robust epileptiform activity with routine visually guided patching may open a window into the different neuronal activities triggering, supporting and stopping epileptiform bursts.

*In vitro* experiments using brain slices are a powerful tool for exploring neuronal pathophysiology in epilepsy. Acute manipulations which can induce epileptiform activity include perfusion with solutions containing elevated potassium (Traynelis and Dingledine, 1988), lowered calcium (Jefferys and Haas, 1982) or zero magnesium (Mody et al., 1987). Such experiments are advantageous as they permit the recording of neuronal network activity with microelectrode techniques whilst perfusing with pharmacological agents (Hill and Greenfield, 2011). In order to perform these recordings, slice tissue must be maintained artificially with sufficient oxygen, heat and supply of nutrients. This is best achieved using an interface or “Haas” style chamber (Haas et al., 1979), in which the slice is covered by a very thin film of oxygenated artificial cerebrospinal fluid (aCSF). This aCSF is continuously circulated and provides a robust environment for recording of experimental oscillations in brain slices (for example, epileptiform oscillations in response to elevated potassium; Traynelis and Dingledine, 1988). Despite the obvious advantages, these Haas-type chambers are not suitable for some types of *in vitro* experiment. Specifically, they are not compatible with the use of high-powered water immersion objective lenses, which are required for visually-guided microelectrode techniques including patch clamp (Hamill et al., 1981). Visually guided patch clamp can be performed using a submerged style chamber, in which the slice rests on a plastic cover slip within a deeper reservoir of aCSF (Li and McIlwain, 1957). However, although this permits the use of high powered objectives, it severely impairs oxygen delivery to the slice, which impacts on the ability to record robust local field potentials (LFPs) (Gloveli et al., 2005; Hájos et al., 2009). Therefore, it is not possible to simultaneously perform LFP recordings of epileptiform bursts and visually-guided patch clamp using conventional *in vitro* chambers. The ability to perform these recordings would permit studies on the contribution of individually identified neurons to network activity. In order to circumvent these shortcomings, numerous studies have reported modifications to the submerged recording conditions in attempts to improve slice viability whilst permitting the use of high powered objectives. Hajos et al (Hájos and Mody, 2009) reported a dual perfusion system that simultaneously perfused both surfaces of the slice. This successfully rescued the deficit in fast oscillations, but required a slow aCSF perfusion rate (~2–3 ml min^−1^) for mechanical stability of the slice. Moreover, suspension of the slice on a net has been suggested to impair high-powered imaging (Hill and Greenfield, 2011).

**Figure 1:**
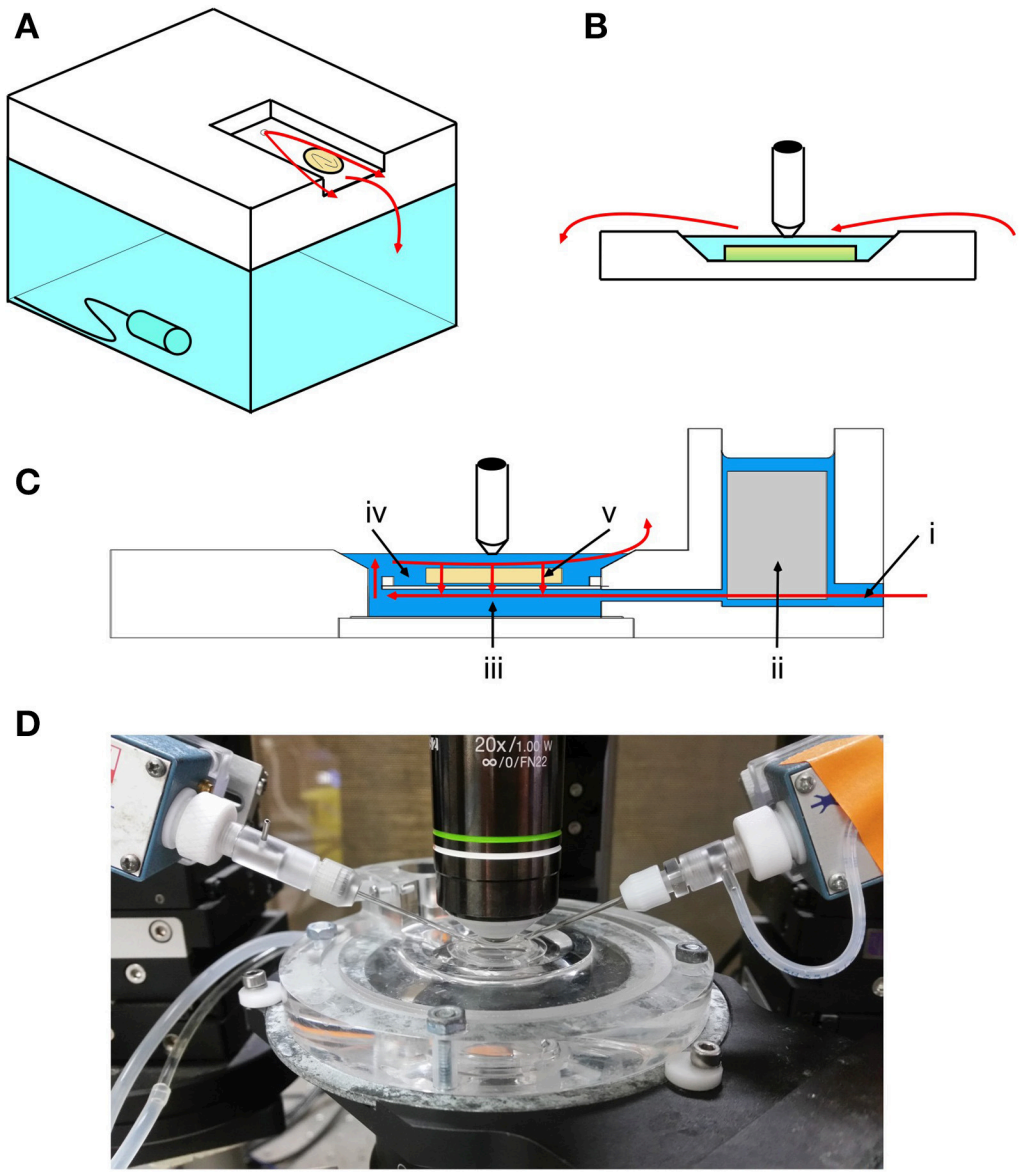
**Overview of interface, submerged and membrane chambers. (A)** In interface conditions, the slice rests on a net, at the interface between perfusing aCSF and air. A thin film of aCSF (flow represented by red arrows in all panels) flows over the slice and further oxygen is added to the local environment by bubbling into the water reservoir below. The slice is warmed by heating the water reservoir. **(B)** In submerged conditions, the slice rests on a plastic coverslip inside a reservoir of aCSF. Oxygen dissolved in the aCSF can only reach the upper surface of the slice, creating a concentration gradient and leading to anoxia in the under surface. Water immersion lenses can be used for high magnification imaging in submerged chambers. **(C)** The membrane chamber aims to combine the technical advantages of the two conventional styles (image modified with permission from original supplied by Sukhvinder Singh Dhanjal, Scientific Systems Design Inc.). The slice rests on a semipermeable membrane. Fast flowing (~16 ml min^−1^) aCSF enters through the inlet (i) and any turbulent flow is buffered by a “scrim” material (ii). aCSF flows quickly underneath the slice (iii) and then relatively slowly over the top of the slice (iv). This creates a Bernoulli effect, which sucks aCSF through the slice and membrane by active flow (v). This efficiently oxygenates the slice and also stabilizes it. **(D)** Photograph showing the membrane chamber in use.

The “membrane chamber” (Hill and Greenfield, 2011) is a novel type of *in vitro* recording chamber which aims to enhance slice viability in submerged conditions (Figure 1). This is achieved in three ways. Firstly, similar to the dual perfusion chamber (Hájos and Mody, 2009), both sides of the slice are perfused. This is achieved by suspending the slice on a flat semi-permeable membrane in the center of the chamber. Secondly, the chamber can support very high flow rates (tested here at ~16 ml min^−1^) whilst retaining mechanical stability. This has the advantage of providing a more rapid turnover of oxygen and is achieved by exploiting the “Bernoulli effect,” where the comparatively fast flow underneath the slice causes it to be effectively sucked down on to the membrane. This effect is determined by the equation (Hill and Greenfield, 2011):

$$\begin{array}{l} \frac{v^{2}}{2}+\psi +\frac{p}{\rho }=\text{constant} \end{array}$$

in which *v* = flow rate, ψ gravitational potential, *p* pressure and ρ density. Ψ and ρ are assumed to be constant and so the reduction in pressure which mediates the Bernoulli effect is proportional to *v*^2^. Thus, the effect is likely to be negated at lower flow rates, though this has not been tested systematically. The Bernoulli effect also creates the third property which enhances oxygen availability—active flow. This causes aCSF to be pulled through the slice, creating a highly effective perfusion. A further advantage of the membrane chamber is that the semi-permeable membrane is both flat and transparent, so the chamber is compatible with both upright and inverted microscopy.

We tested whether the membrane chamber was able to provide a suitable *in vitro* experimental environment to simultaneously model epileptiform activity and record from visually-identified neurons using patch clamp. We applied this to explore the cellular correlates of network high frequency activity (HFA) in the epileptic hippocampus. HFA consists of activity in the 100–500 Hz frequency band and is strongly linked to epilepsy (Bragin et al., 1999; Jiruska et al., 2010a; Jefferys et al., 2012). We showed that the membrane chamber was capable of supporting more robust HFA than conventional submerged chambers. We demonstrated the visualization and patch clamp of fluorescent YFP-tagged interneurons, during ongoing epileptiform LFP activity. We then applied this technique to explore the phase relationships between interneuron firing and field HFA. Fast ripple HFA is a biomarker for epileptic tissue (Bragin et al., 1999) and can be recorded *in vivo* using the chronic intrahippocampal tetanus neurotoxin (TeNT) model (Jiruska et al., 2010b). We show that in the membrane chamber this activity can also be modeled in *ex vivo* slices from TeNT-treated rats. This is consistent with a recent study on neocortex which suggests that features of the chronic epileptic condition are maintained in *ex vivo* slice preparations (Serafini et al., 2016). The cellular correlates of fast ripple activity are unknown, and the membrane chamber provides a new type of recording system in which they could be easily and conveniently probed in brain slices.

## Methods

### Ethical approval

All experimental procedures were carried out in accordance with the Animals (Scientific Procedures) Act 1986 and were approved by the Institutional Biomedical Ethical Review Sub-Committee at the University of Birmingham.

### Slice preparation

Adult VGAT-Venus A rats (Uematsu et al., 2008) of both genders were anesthetized with 0.24 mg/kg medetomidine hydrochloride and 58.2 mg/kg ketamine via intraperitoneal injection prior to cardiac perfusion with sucrose aCSF (in mM: 205 sucrose, 2.5 KCl, 26 NaHCO_3_, 1.2 NaH_2_PO_4_.H_2_O, 10 glucose, 5 MgCl_2_, 0.1 CaCl_2_) at a rate of 13.3 ml min^−1^. Perfused brains were quickly dissected into ice cold sucrose aCSF and 300 μm thick horizontal slices were prepared using a 7000 smz integraslice (Campden Instruments, Loughborough, UK). Slices were stored at room temperature inside a holding chamber on an interface between oxygenated aCSF (in mM: 10 glucose, 125 NaCl, 3 KCl, 26 NaHCO_3_, 1 MgCl_2_, 1.25 NaH_2_PO_4_.H_2_O and 2 CaCl_2_) and 95% O_2_/5% CO_2_ for at least 1 h, then transferred at random to either interface, conventional submerged or membrane chamber conditions.

### TeNT surgery

For *ex vivo* modeling of chronic epilepsy, rats were injected with unilateral intrahippocampal TeNT (2.5 ng in 1 μl PBS with 2% BSA; List Biological Laboratories Inc., California, USA; (Mellanby et al., 1977; Jiruska et al., 2010b). Stereotaxic injections were targeted to the CA3 region of the right ventral hippocampus (co-ordinates relative to bregma: AP −4.3 mm, ML +4.4 mm, DV −7.5 mm). Injections used a 1 μl syringe (model no. 7001 KH, Hamilton Company, Nevada, USA), controlled by a microinjection pump (KDS 311CE, kd Scientific, RoYem Scientific Limited, UK) to give a rate of 0.2 μl min^−1^. The needle was left in place for 5 min after the injection, in order to minimize backflow along the injection track. Surgery was carried out under isofluorane anesthesia (1.5–2.5% in oxygen at 1 l min^−1^) and continuously monitored using absence of pedal reflex as a marker. Rats were given 5 ml glucose/saline solution and 0.1 mg kg^−1^ buprenorphine, both subcutaneously, at the beginning of surgery. Slices ipsilateral to the injection site were used for *ex vivo* recordings. Video recordings confirmed that all rats exhibited generalized seizure activity prior to slice preparation.

### Membrane chamber recordings

Slices recorded in the membrane chamber (Scientific Systems Design Inc., Ontario, Canada; supplied by Digitimer, Welwyn Garden City, UK) were perfused with oxygenated aCSF, heated to 32°C, at ~16 ml min^−1^ (close to the maximum flow rate that was initially tested in Hill and Greenfield, 2011). Slices were equilibrated in these conditions for at least 10 min before recording. A new membrane insert (plastic inserts: Digitimer, part number S.MCK10; membrane: SnakeSkin dialysis tubing (10,000 MWCO), Fisher Scientific UK, Loughborough, UK) was prepared at the start of each experimental day. The chamber was also tested using a net in place of the semi-permeable membrane, however this impaired imaging and caused the slice to be dragged into the spaces in the net, compromising slice physiology and stability. Slices were visualized with an FV1000 confocal microscope (Olympus UK Ltd., Southend on Sea, UK). Fluorescence imaging used the appropriate confocal laser and Olympus Fluoview software (Olympus UK Ltd.). Venus fluorescence used 515 nm for excitation and 530–570 nm for emission (Uematsu et al., 2008). Alexa 647 used 635 nm for excitation and 650–750 nm for emission. Both LFP and single-cell recordings used borosilicate glass micropipettes (4–7 MΩ). The extracellular pipette was filled with normal aCSF and placed into CA3b stratum pyramidale. The patch pipette was filled with internal solution (in mM: 130 KMeSO_4_, 8 NaCl, 10 HEPES, 2 Mg-ATP, 0.3 Na-GTP; pH 7.3 with KOH; osmolarity 280 mOsm),with 200 nM Alexa 647 hydrazide (Life Technologies, Paisley, UK). Patch recordings were targeted to interneurons using Venus as a visual biomarker. Signals were amplified using an Axoprobe 1A patch clamp amplifier (Molecular Devices, California, USA) and, for variable gain, a NL106 AC-DC Amplifier (Digitimer, Welwyn Garden City, UK). All signals were filtered at DC-2 kHz using a NL125/126 filter (Digitimer). Signals were digitized at 5 kHz using a Micro 1401 (Cambridge Electronic Design, Cambridge, UK) and recorded using Signal or Spike 2 (both Cambridge Electronic Design). Epileptiform activity was induced by raising [K^+^]_o_ to 9 mM.

### Submerged recordings

LFP recordings made in the conventional submerged chamber used the same conditions as for the membrane chamber, with the exception that aCSF was circulated at a rate of 4–6 ml min^−1^, roughly the maximum supported by this type of chamber without resulting in slice mechanical instability (Hájos and Mody, 2009).

### Interface recordings

For interface recordings, slices were perfused with oxygenated aCSF, heated to 32°C, at a rate of 4–6 ml min^−1^. Slices were left to equilibrate to these conditions for 1 h prior to recording. After this time, a glass micropipette (4–7 MΩ resistance) was placed in hippocampal CA3b SP. Signals were amplified using an Axoprobe-1A amplifier (Molecular Devices), digitized at 5 kHz with a Power 1401 (Cambridge Electronic Design, Cambridge, UK) and recorded using Spike2 (Cambridge Electronic Design). Epileptiform activity was induced by raising [K^+^]_o_ to 8 or 8.5 mM. Higher potassium concentrations, as used in the other two chambers, often caused spreading depression in the interface condition. This is likely due to local accumulation of extracellular potassium ions (Obrenovitch and Zilkha, 1995).

### Data analysis

Power, peak frequency and oscillation length analyses were performed using Spike2 (CED). Time data were converted to the frequency domain using fast Fourier transform (FFT length: 4096, Hanning window) to produce a power spectrum. Total HFA power was measured as the sum of power between 100 and 500 Hz. Peak HFA frequency was measured as the frequency with the greatest power between 100 and 500 Hz. To estimate average burst length, raw data were bandpass filtered at 100–500 Hz (FIR digital filter, length = 679) and individual HFA cycles were detected as negative troughs below a threshold of 7 × baseline standard deviation. An average waveform was processed for these cycles. The average burst length is the number of troughs on the average waveform which exceed 7 × baseline SD of the average waveform. For the observation of fast ripples in *ex vivo* slices, raw traces were bandpass filtered at 300–500 Hz (FIR digital filter, length = 679). Power spectra were created as above. To analyse phase relationships between interneuron firing and HFA, LFP events were detected as above and interneuron action potentials detected as peaks greater than 7 × baseline SD in the single-cell traces. A custom MatLab (Mathworks, Massachusetts, USA) script was use to process phase relationships and to test them for significance with Rayleigh statistics.

### Statistics

Statistical tests were performed using IBM SPSS Statistics (version 22.0, IBM Corp., USA). Data were tested for normality using the Kolmogorov Smirnov test. When comparing activity recorded in each chamber, three statistical comparisons (HFA power, HFA peak frequency and average cycle length) were made from the same data. Therefore, α was corrected to 0.05/(3–1) = 0.025. Statistical power was calculated using G^*^Power 3.1.9.2 (Faul et al., 2007).

## Results

### Comparison of epileptiform activity in the membrane chamber with other conditions

**Figure 2:**
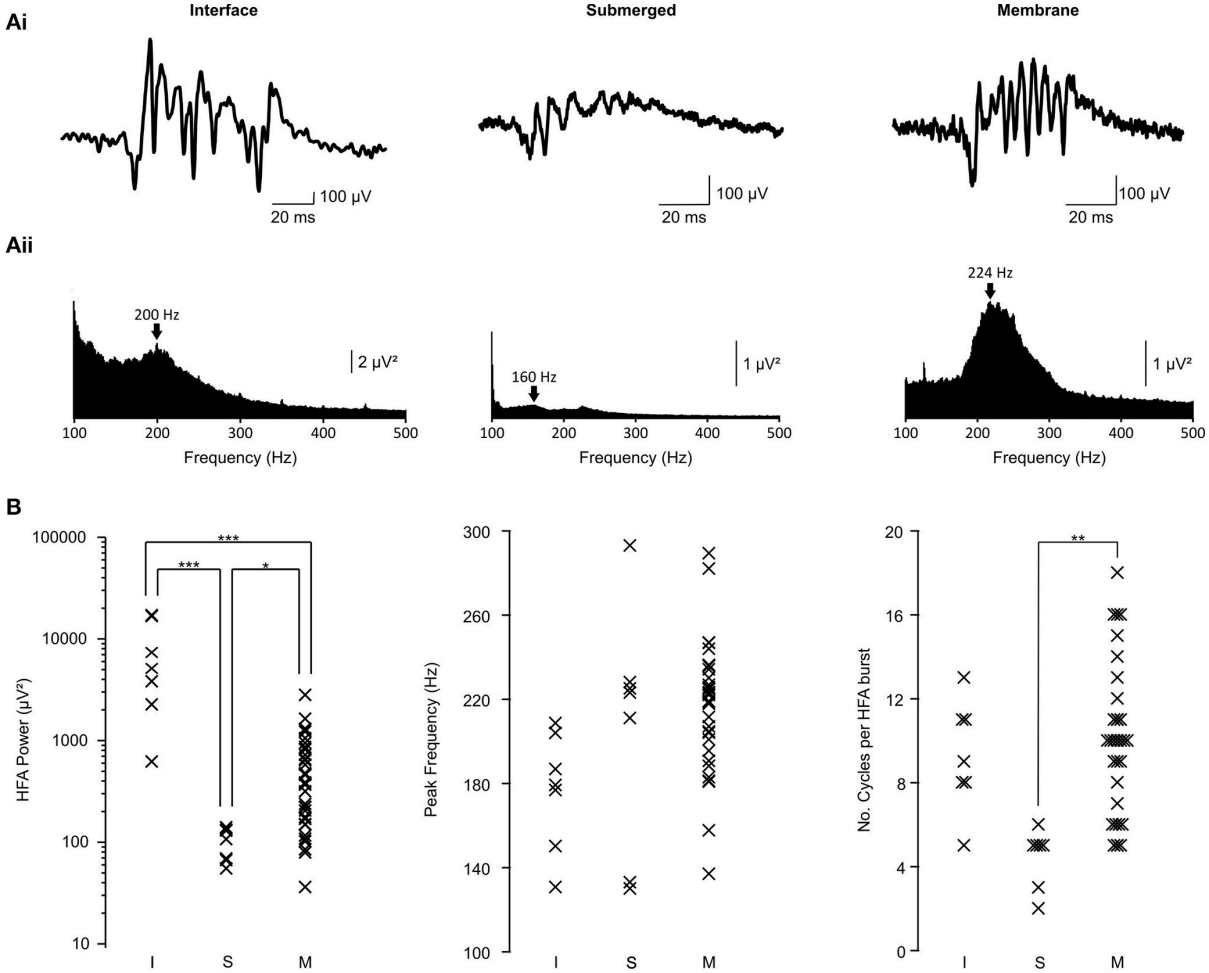
**HFA properties in interface, submerged and membrane chambers. (Ai)** Raw traces showing a representative burst of high frequency activity in each chamber type. The submerged and membrane traces are presented on the same amplitude scale. All traces are presented on the same time scale. **(Aii)** Power spectra derived from the traces shown in **(Ai)**. Arrows indicate the peak frequency within HFA band on each trace. Submerged and interface power spectra are presented on the same scale. **(B)** Population data showing all measured parameters for each slice. Data are separated into interface (I; *n* = 7 slices from 6 animals), submerged (S, *n* = 7 slices from 5 animals) and membrane (M, *n* = 33 slices from 24 animals). For power: ^***^p < 0.0005; ^*^ *p* < 0.025; ANOVA with Bonferroni *post-hoc* correction. For number of cycles: ^**^ *p* < 0.005; Kruskal Wallis test.

Our hypothesis was that using the membrane chamber in place of more conventional recording chambers would provide the ability to simultaneously model epileptic network activity and record from individual visually-identified neurons. Thus, we began by testing the ability of the membrane chamber to support epileptiform network activity, using the elevated potassium model *in vitro* (Traynelis and Dingledine, 1988). We applied high extracellular potassium to brain slices distributed randomly between membrane, submerged and interface chambers. High frequency activity (>~100 Hz) is a prominent feature of this model and is strongly associated with epilepsy (Bragin et al., 1999; Jiruska et al., 2010a; Jefferys et al., 2012). We therefore measured the power, frequency and number of cycles per burst of HFA in each chamber and used these properties to compare between each environment (Figure 2).

HFA power was recorded as the total power between 100 and 500 Hz, in line with most conventional estimates of the frequency band. In slices recorded in the membrane chamber (Figure 2, right column; *n* = 33 slices from 24 animals), power in this frequency band was 579 ± 102 μV^2^. This was significantly higher than in the submerged chamber (Figure 2, middle column; 100 ± 14 μV^2^; 7 slices from 5 animals; one way ANOVA with Bonferroni *post-hoc* correction on log-transformed data; *p* = 0.006), but significantly lower than slices in the interface chamber (Figure 2, left column; 7598 ± 2552 μV^2^; 7 slices from 6 animals; *p* = 4 × 10^−7^). Therefore, slices in the membrane chamber generated more powerful activity than those in conventional submerged conditions, but this was not as great as could be elicited in interface conditions. However, it must also be considered that HFA power in interface conditions was considerably more variable (variances – interface: 45597581 μV^4^, submerged: 1293 μV^4^, membrane: 343657 μV^4^; variance ratios (F_(df1, df2)_); α = 0.025: interface vs. submerged—35265 [*F*_(6, 6)_ = 5.8198]; interface vs. membrane—133 [*F*_(6, 32)_ = 2.8667]; submerged vs. membrane—266 [*F*_(32, 6)_ = 5.065]) and that non-physiological factors, such as shunting of currents into aCSF, could play a role (see discussion). The reduced variability in the membrane chamber confers greater reproducibility in such experiments and is an advantage over interface conditions.

We then compared the dominant frequency of HFA between the three chambers (Figure 2B middle column). In the membrane chamber, peak HFA frequency was 217 ± 5 Hz (33 slices from 24 animals). Peak frequency in the other two chambers was 177 ± 10 Hz for interface (7 slices; 6 animals) and 206 ± 22 Hz (7 slices; 5 animals) for submerged. None of the differences observed were statistically significant at α = 0.025 (one way ANOVA with Bonferroni *post-hoc* correction; membrane vs. submerged *p* = 1; membrane vs. interface *p* = 0.048; interface vs. submerged *p* = 0.47). The lack of difference between the chambers in terms of the frequency of the K^+^-induced hippocampal HFA suggests the phenomenon is similar in all three.

Finally, we compared the average number of cycles per HFA burst (Figure 2B right column). This was an important parameter to test because it has previously been suggested that a limited oxygen supply to the slice, as happens in conventional submerged chambers, leads to truncated oscillations (Gloveli et al., 2005; Hájos et al., 2009). Oscillations in the membrane chamber lasted for 10.0 ± 0.6 cycles. This was no different from the interface chamber (9.3 ± 1.0 cycles; Kruskal Wallis test; *p* = 1) but significantly greater than the submerged chamber (4.4 ± 0.5 cycles; *p* = 0.001). This finding was key because it indicated that the membrane chamber could support much more prolonged and robust HFA than the conventional submerged condition. Further, the membrane chamber produced oscillations with a similar length to those in interface conditions, in which oxygen is readily available. Interface chamber oscillation length was not significantly longer than submerged conditions at α = 0.025 (*p* = 0.027).

### Simultaneous visually-guided patch clamp and epileptiform field recordings in the membrane chamber

**Figure 3:**
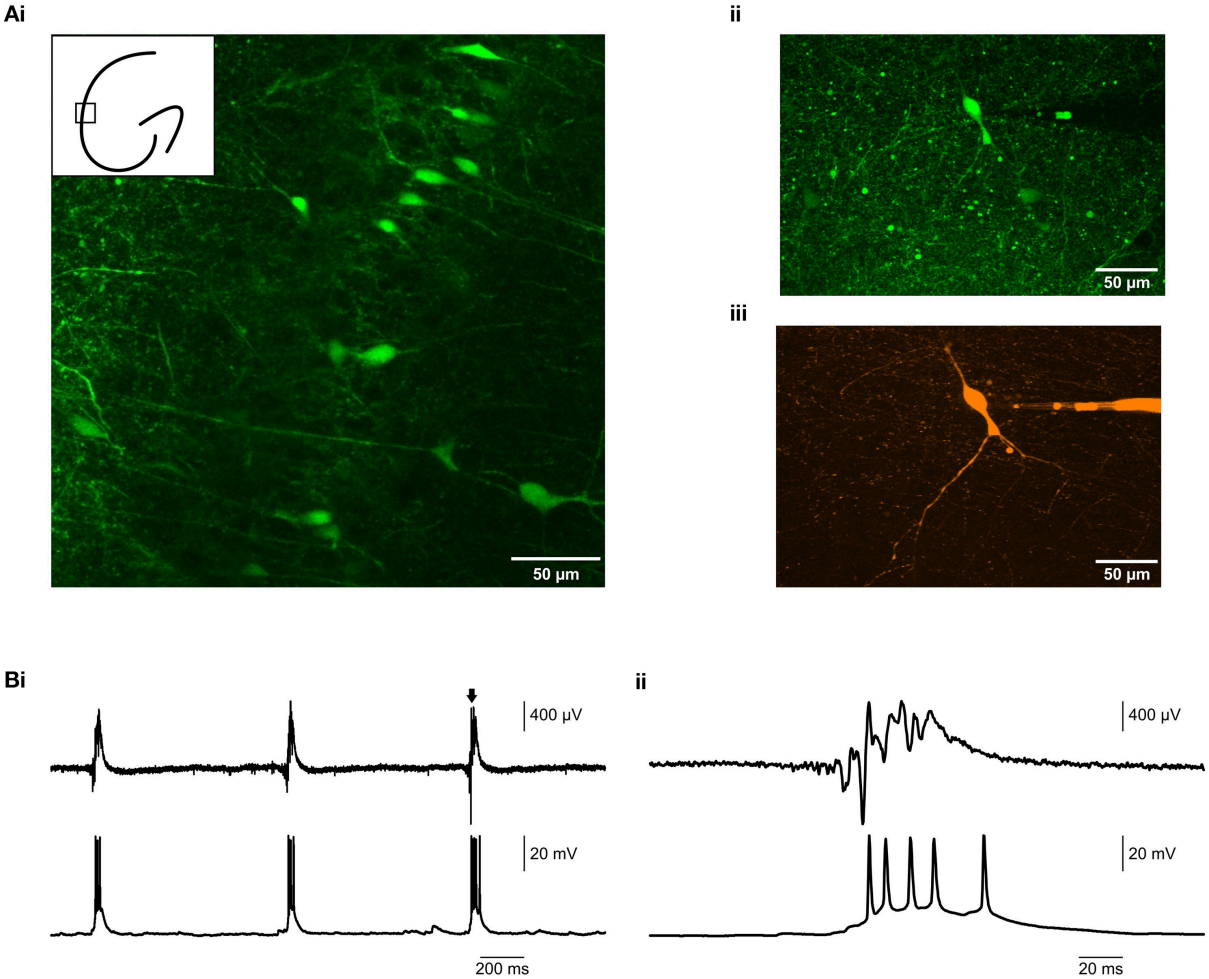
**Simultaneous visually-guided patch clamp and field recording in 9 mM K^**+**^. (Ai)** 20 × image of a brain slice from a VGAT-Venus A rat (Uematsu et al., 2008), showing fluorescently labeled interneurons in hippocampal CA1. The inset cartoon roughly shows the imaged region. **(Aii)** 40x image of an individually targeted interneuron in hippocampal CA3b. **(Aiii)** The same interneuron, filled with 200 nM Alexa 647 hydrazide for improved visualization. **(B)** Epileptiform activity (LFP, upper traces) was induced by 9 mM K^+^, whilst simultaneously recording the firing of a targeted interneuron in current clamp mode (lower traces).**(Bii)** is an expanded view of the burst indicated by the arrow in **(Bi)**.

Having established that it was possible to record robust epileptiform activity in the membrane chamber, we tested whether the chamber was compatible with patch clamp recordings from visually identified neurons. We used brain slices from VGAT-Venus A rats (Uematsu et al., 2008), in which interneurons are marked with a YFP tag. Using fluorescence microscopy, we were able to identify and visualize interneurons for targeted recording (Figure 3A). We subsequently patch clamped the identified neurons and recorded their activity in current clamp mode (Figure 3B), thus demonstrating that the membrane chamber maintained the technical advantages of conventional submerged chambers. Further, this could be performed simultaneously with epileptiform field recordings. Therefore, the membrane chamber provided an *in vitro* environment in which the contribution of single neurons to epileptiform field activity could be probed.

### Interneuron firing during *in vitro* high frequency activity

**Figure 4:**
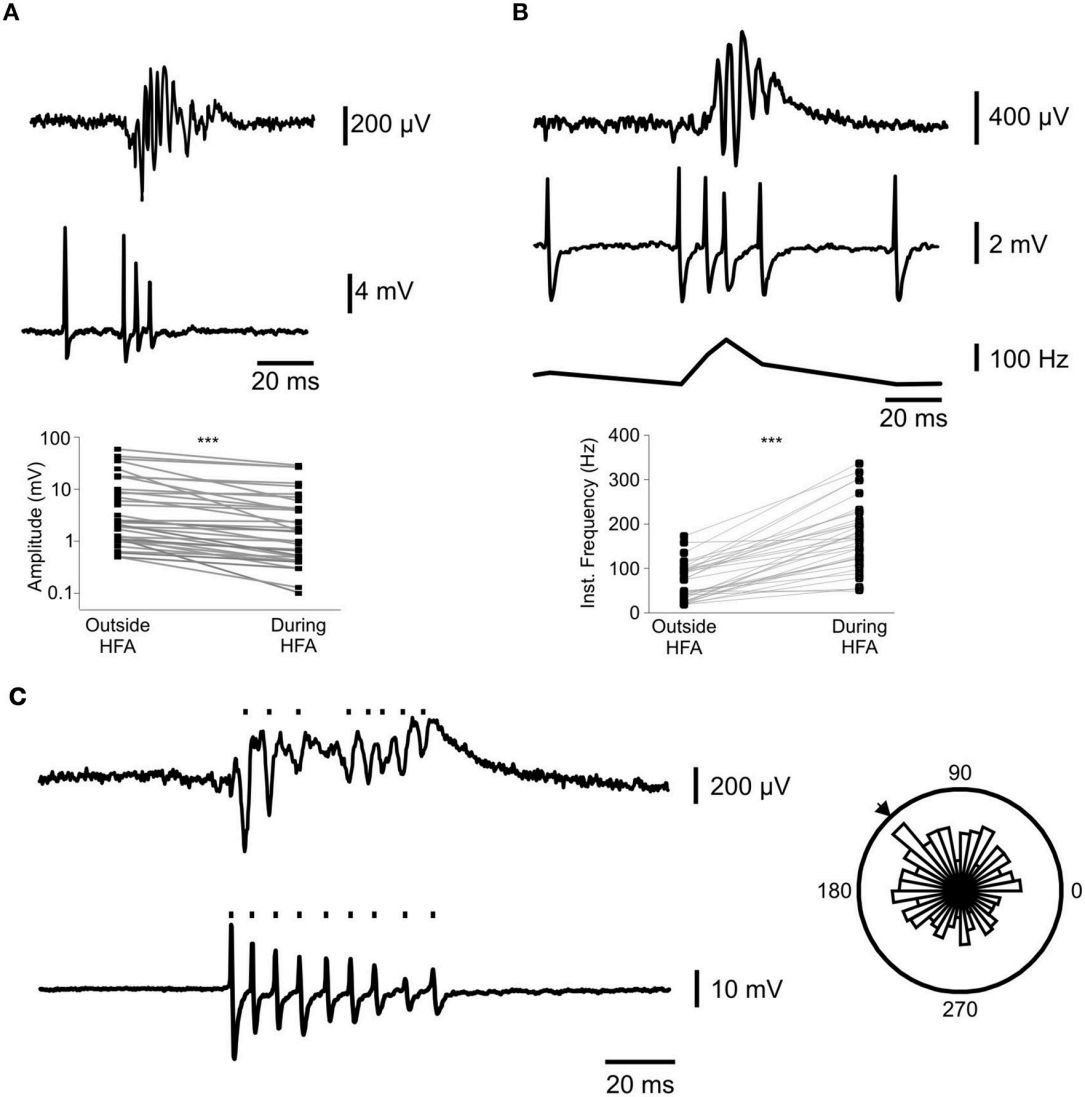
**Interneuron firing has high frequency and low amplitude during bursts and can participate in HFA. (A)** shows a representative progressive decrease in action potential amplitude in (loose patch configuration (middle panel)) during the simultaneous burst in local field potential recordings (top panel). The scatterplot (bottom panel) shows the baseline action potential amplitude paired to the minimal amplitude during an epileptiform burst for 34 interneurons (^***^p = 3.6 × 10^−7^; Wilcoxon Signed Ranks test). **(B)** represents the increase in interneuron firing frequency (ii) during epileptiform bursts (i). Panel (iii) shows the instantaneous frequency of the action potentials in (ii). (iv) shows the paired increases in firing frequency during epileptiform bursts for 34 interneurons (^***^p = 3.6 × 10^−7^, Wilcoxon Signed Ranks test). **(C)** shows the detection of HFA (upper trace) and action potential (lower trace) events. Rose plot shows a significant (Rayleight test *p* < 0.05) relationship between the two. The arrow denotes the mean phase lag between the signals.

We next used the membrane chamber to explore the contributions of individual interneurons to high frequency activity *in vitro*. We recorded from 36 fluorescently labeled interneurons, whilst simultaneously recording LFP activity induced by 9 mM K^+^. In the transgenic rat, the fluorescent label was co-expressed with VGAT, a protein ubiquitous to all interneurons, and so it was impossible to distinguish between the multiple sub-types which are present in the hippocampus (Klausberger and Somogyi, 2008); see also Figure 3A). Nevertheless, we observed effects which were common to all interneurons examined. Firstly, there was a progressive decrease in the amplitude of interneuron action potentials during field HFA bursts (Figure 4A). Minimal action potential amplitude during bursts was 54 ± 4% of the initial amplitude (Wilcoxon Signed Ranks test; *p* = 3.6 × 10^−7^). Secondly, interneuron firing frequency markedly increased during field bursts (Figure 4B), from 52 ± 8 Hz to 155 ± 13 Hz (Wilcoxon Signed Ranks test; *p* = 3.6 × 10^−7^). Physiological ripple activity is involved in learning and memory and is thought to be paced by recurrent inhibition from fast-spiking interneurons (Ylinen et al., 1995). It has been hypothesized that the same could be true for epileptiform HFA (Jiruska et al., 2010c). We therefore explored the phase relationships between interneuron firing activity and field HFA. Circular statistics (Fisher, 1995) revealed significant relationships between HFA and single-cell spiking in 15 interneurons (7 in stratum oriens, 3 stratum pyramidale and 5 stratum radiatum; Figure 4C).

### *Ex vivo* models of chronic epilepsy using the membrane chamber

Given that the membrane chamber provided an environment suitable for robust and reliable acute models of epilepsy, we then tested if the chamber would support spontaneous activity in *ex vivo* slices taken from chronically epileptic rats. This type of activity would be a more representative model of “real” epilepsy, as it does not rely on large artificial perturbations of ion concentrations to induce epileptiform activity.

**Figure 5:**
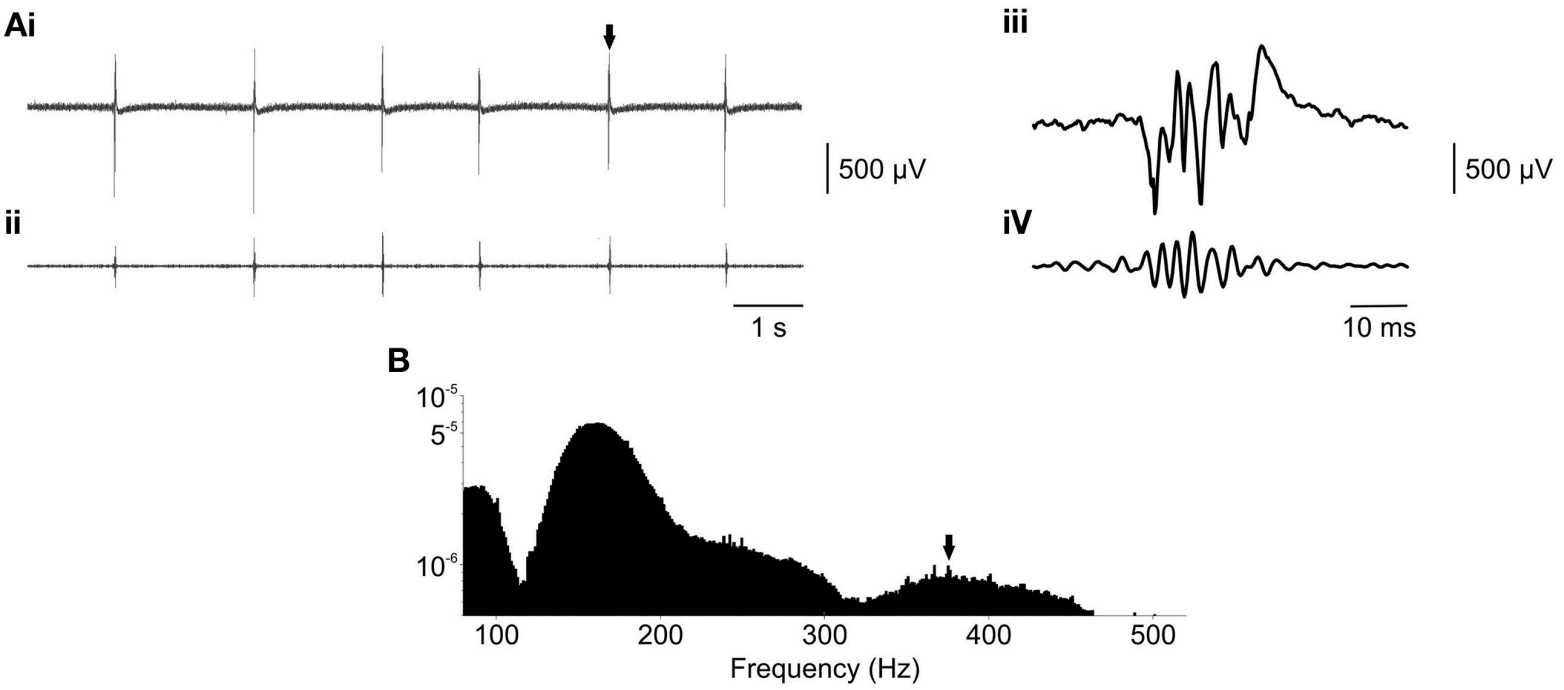
**Fast ripples can be modeled in the membrane chamber using ***ex vivo*** slices. (Ai)** Raw epileptiform fields recorded *ex vivo*, in a brain slice from a TeNT-treated rat, in the presence of 6 mM K^+^. **(Aii)** Raw data from **(Ai)**, bandpass filtered between 300 and 500 Hz, revealed the presence of fast ripple oscillations. This was confirmed by examination of the waveforms on a shorter time base (**Aiii**, raw and **Aiv**, 300–500 Hz) and power spectral analysis **(B)**, power calculated by fast Fourier transform, length = 4096, and plotted on a logarithmic scale) which revealed a separate peak in the fast ripple band, as indicated by the arrow.

Although we did not observe spontaneous activity in any of the 13 slices recorded, seizure activity was induced in 9 out of 13 slices by a modest elevation of K^+^, to 6 mM (Figure 5). In comparison, this concentration only induced seizure activity in 1 out of 5 control slices. This difference is not significant, though statistical power is compromised by the low control sample size (Fisher's exact test, *p* = 0.088, statistical power = 0.44). Therefore, we cannot conclude that *ex vivo* slices from TeNT treated animals were more prone to seizure activity than naïve slices.

However, power spectral analysis (Figure 5B) revealed that 4 out of 13 slices exhibited >250 Hz fast ripple activity. This phenomenon was distinct from the ripple-band (~100–250 Hz) activity seen in the acute high potassium model and perhaps reflects the chronically epileptic nature of the *ex vivo* tissue. Fast ripple activity had a peak frequency of 347 ± 28 Hz. Calculation of the fast ripple band power was complicated by frequency overlap with larger spectral peaks in the ripple band. Therefore, fast ripple power was estimated between 300 and 500 Hz (clear of ripple band activity) at 84 ± 16 μV^2^. The presence of spectral peaks in the fast ripple frequency band, which were not observed in control slices, suggested that epileptiform activity generated in *ex vivo* slices from TeNT treated rats may better model the chronic condition.

## Discussion

We tested whether the membrane chamber provided a viable environment for the simultaneous recording of single-cell activity in patch clamp and epileptiform field activity. This would provide a novel experimental method that permits the contribution of individually identified neurons to epileptiform field activity to be examined. We first used the acute elevated potassium model of epilepsy to show that epileptiform activity in the membrane chamber was substantially more robust than in an ordinary submerged chamber. We then demonstrated the use of the membrane chamber for targeted patch clamp of fluorescent neurons. This was performed simultaneously with the recording of epileptiform fields. We used this novel technique to explore the relationships between interneuron firing and epileptiform HFA, revealing significant correlations between the two. Finally, we explored whether the membrane chamber could be used to better model epilepsy by using *ex vivo* slices from chronically epileptic animals. We did not observe spontaneous epileptiform activity with 3 mM K^+^, but these slices often had a lower seizure threshold and also featured power spectral bands unique to chronic epilepsy.

### Comparison with interface and submerged chambers

We first characterized epileptiform activity in the hippocampus using the elevated potassium model. We used high frequency activity, a prominent feature of epileptic waveforms in this model, to compare the three conditions. Specifically we measured the power, frequency and number of cycles in each oscillation (Figure 2). We hypothesized that the membrane chamber would perform comparably with the interface chamber whilst generating substantially more robust epileptiform LFPs than the submerged chamber. The key finding was that the length of oscillations (measured by number of cycles) was similar in membrane and interface chambers, with significantly longer oscillations in membrane than in submerged. It has been shown that fast oscillations are truncated in the submerged chamber (Gloveli et al., 2005; Hájos et al., 2009), owing to reduced oxygen availability to the slice. Similarly, HFA generated by slices in the membrane chamber was more powerful than in the submerged chamber These findings provide evidence that the properties of the membrane chamber (active flow through the slice, fast flow rate and perfusion of both slice surfaces) overcome the limited oxygen supply of normal submerged chambers and enable the generation of more robust oscillations in brain slices. It is noteworthy that HFA in the membrane chamber was considerably less powerful than the interface condition, despite having similar frequency and oscillation length. This could be due to shunting of recorded potentials into aCSF, in which resistivity is around five times lower than in neuropil (Fox et al., 2004). This effect is considerably greater in the membrane chamber, where there is a deeper reservoir of aCSF, than it is in the interface chamber, where the slice is perfused by a thin film of aCSF. The three chambers produced oscillations with similar frequencies, suggesting that they were the same phenomenon each time.

### Simultaneous field recording and patch clamp

We showed that the membrane chamber could support epileptiform activity which was comparable to interface chambers and considerably more robust than submerged chambers. We then demonstrated that the membrane chamber retained the technical advantages of conventional submerged conditions—specifically the ability for visually-guided patch clamp recording (Figure 3). Using fluorescence confocal microscopy, we observed fluorescent interneurons in brain slices in the membrane chamber. We were able to resolve the somata and thicker projections of these neurons. We then recorded from the visually identified cells with patch clamp. Both confocal imaging and patch clamp are techniques which require the slice to be mechanically stable. The design of the membrane chamber creates a Bernoulli effect underneath the slice, as has previously been determined experimentally (Hill and Greenfield, 2011). This is caused by a comparatively fast flow of perfusate underneath the membrane and a slower flow over the top and the result is to effectively suck the slice on to the membrane. This effect created sufficient slice stability at a flow rate of 16 ml min^−1^. There was no requirement to use any form of slice harp or weight. Therefore, the membrane chamber combines the advantages of both interface (robust LFP recordings) and submerged (visually guided patch clamp) recordings to create a novel environment for *in vitro* epilepsy modeling.

### Interneuron firing during epileptiform HFA

We used this novel technique to begin to explore how interneurons may participate in epileptiform network activity. We observed a progressive decrease in action potential amplitude and an increase in firing frequency which was associated with field discharges (Figure 4). This is comparable with other studies (Karlócai et al., 2014), in which interneurons increased their firing rate during interictal discharges. In experiments recorded in whole cell current clamp configuration, we observed large EPSPs in interneurons during field bursts (see Figure 3B). Thus, it is likely that the progressive decrease in action potential amplitude is caused by a gradual increase in V_m_ toward E_Na_, meaning that the interneurons require a smaller influx of sodium (which underlies the depolarizing phase of the action potential) to reach E_Na_. We then explored the phase relationships between interneuron firing and individual HFA cycles. It has been hypothesized that epileptiform HFA rhythms are synchronized by feedback inhibition from interneurons (Jiruska et al., 2010c). It was not possible to classify individual sub-types of interneurons (Klausberger and Somogyi, 2008) in these experiments, but recorded cells were categorized roughly by their laminar distribution in the hippocampus, with several observations common to all interneurons, despite their heterogeneity. The most pertinent result was that 15 interneurons showed firing which was significantly correlated with field HFA cycles (Figure 4C). This implied a reciprocal interaction between the two signals and demonstrates interneuronal involvement in epileptiform HFA.

### *Ex vivo* modeling of chronic epilepsy

Given that the membrane chamber supported the generation of robust epileptiform activity in brain slices in response to elevated potassium, we then explored patterns of epileptiform activity in chronically epileptic networks, using *ex vivo* slices from rats treated with intrahippocampal TeNT. It has been suggested that features of chronic epilepsy are retained in *ex vivo* neocortical slices (Serafini et al., 2016). All of the rats used for tissue preparation were confirmed by video recording to have experienced spontaneous seizures *in vivo* in response to TeNT injection. However, none of the 13 slices tested generated spontaneous epileptiform activity in normal aCSF. Previous studies found that spontaneous epileptiform activity is not present (Jordan and Jefferys, 1992) or occurs only in a minority of TeNT-treated hippocampal slices (Jefferys, 1989) in interface conditions, probably depending on the exact ddose and potency of TeNT used. There could be multiple other explanations for the lack of spontaneous epileptiform activity in our experiments. During the slicing procedure, the brain networks which generated spontaneous seizures *in vivo* could have been severed, stopping them from activating spontaneously. Similarly, the neuronal networks within a 300 μm brain slice could be too small to generate this activity in normal aCSF with physiological ion concentrations. It may be more appropriate to use thicker slices to record spontaneous *ex vivo* epileptic activity, but this was not suitable in the current study as 300 μm was considered the maximum thickness amenable to patch clamp recordings. Another factor affecting seizure generation could be temperature. During *in vivo* seizures, the brain is at 37°C—body temperature in the live rat. The *ex vivo* recordings were made in slices held at 32°C, and the reduced temperature could lead to slower kinetics of seizure-generating components. Slice recordings are conventionally made at this temperature owing to the inability of oxygen supply to keep up with metabolic demand. It is possible that the increased oxygen supply afforded by the membrane chamber could facilitate recording at higher temperatures, though we did not test this systematically.

Nevertheless, epileptiform activity was induced in 9 out of 13 TeNT treated slices using a modest elevation of K^+^ concentration to 6 mM. This concentration was usually sub-threshold in naïve slices (where concentrations ~8.5 mM are typically required; Traynelis and Dingledine, 1988) and probably reflects a reduced seizure threshold in the *ex vivo* slices. The critical finding was that 4 out of 13 slices generated epileptiform activity which featured oscillations in the fast ripple (>250 Hz) frequency band. Fast ripples are unique to chronically epileptic tissue (Bragin et al., 1999) and are considered a biomarker for the condition. Previous work in our lab showed that fast ripple activity is a feature of the intrahippocampal TeNT model *in vivo* (Jiruska et al., 2010b) and, crucially, we now show that the phenomenon is retained in *ex vivo* slice preparations. This suggests that many of the features of the chronic network were retained *ex vivo*, providing a slice model which more accurately reflected the real condition. This could represent a powerful tool, conferring the ability to easily study chronically epileptic networks at a cellular level.

The membrane chamber is a new type of *in vitro* recording chamber which shares the technical advantages of submerged chambers whilst supporting considerably more robust epileptiform field activity. We used this novel technique to show that interneuron firing can be closely correlated with cycles of network HFA. Finally, we showed that pathological fast ripple activity, unique to chronic epilepsy, could be produced *ex vivo* in the membrane chamber. This provides a promising technique for future studies into the cellular correlates of fast ripple activity.

## Author contributions

GM designed, performed and analysed the experiments. AP co-performed and analysed experiments on HFA activity in the submerged chamber. PJ co-analysed the phase relationship data. AP, PJ and JJ conceived the study, provided leadership, experimental design and project management. All authors discussed the results and wrote the manuscript.

### Conflict of interest statement

The authors declare that the research was conducted in the absence of any commercial or financial relationships that could be construed as a potential conflict of interest.
